# A remarkable case of mosaic parapatry in millipedes

**DOI:** 10.3897/zookeys.156.1893

**Published:** 2011-12-20

**Authors:** Robert Mesibov

**Affiliations:** 1 Queen Victoria Museum and Art Gallery, Launceston, Tasmania 7250, Australia

**Keywords:** Diplopoda, Polydesmida, Dalodesmidae, millipede, Australia, Tasmania, parapatry, boundary estimation, Delaunay triangulation

## Abstract

The parapatric boundary between *Tasmaniosoma compitale* Mesibov, 2010 and *Tasmaniosoma hickmanorum* Mesibov, 2010 (Polydesmida: Dalodesmidae) in northwest Tasmania was mapped in preparation for field studies of parapatry and speciation. Both millipede species can be collected as adults throughout the year, are often abundant in eucalypt forest and tolerate major habitat disturbance. The parapatric boundary between the two species is ca 100 m wide in well-sampled sections and ca 230 km long. It runs from sea level to 600-700 m elevation, crosses most of the river catchments in northwest Tasmania and several major geological boundaries, and one portion of the boundary runs along a steep rainfall gradient. The location of the boundary is estimated here from scattered sample points using a method based on Delaunay triangulation.

## Introduction

Millipedes commonly form lineage mosaics (Mesibov 2003c), in which each species has a discrete range that overlaps very little or not at all with the ranges of other species in the same genus (Shelley 1990a, 1990b). Tasmanian examples in the dalodesmid Polydesmida are found in the genera *Atrophotergum* Mesibov, 2004 (Mesibov 2004), *Dasystigma* Mesibov, 2003 (Mesibov 2003a), *Gasterogramma* Jeekel, 1982 (Mesibov 2003b), *Lissodesmus* Chamberlin, 1920 (Mesibov 2006) and *Tasmaniosoma* Verhoeff, 1936 (Mesibov 2010).

It is not yet known how the tight parapatry seen in these mosaics originates and is maintained (Mesibov 2003c). What is apparent is that parapatric boundaries between millipede species are not necessarily congruent with environmental boundaries. In Tasmania the boundaries cross rivers and traverse different underlying geologies, soil types, local climates and vegetation types.

I document below a remarkable parapatric boundary of the ‘environmentally incongruent’ kind between two congeneric millipedes in northwest Tasmania. I also demonstrate a simple analytical method for estimating and visualising the location of a parapatric boundary from point localities.

## Materials and methods

### Millipede species

The endemic Tasmanian species *Tasmaniosoma compitale* Mesibov, 2010 and *Tasmaniosoma hickmanorum* Mesibov, 2010 were named, described and provisionally mapped in Mesibov (2010). Both are ca 15 mm long as adults. The two species are very similar in appearance, but live and freshly preserved males and females can usually be identified by a difference in colour: *Tasmaniosoma hickmanorum* are red-brown, while *Tasmaniosoma compitale* are yellow-brown with a distinct light patch just under the lateral margin of each paranotum (Fig. 1 in Mesibov 2010). Unfortunately, live colouring in both species is somewhat variable, and some females cannot be confidently assigned to species. For this reason, locations for females not associated with males are marked separately in the map Figures, below, and parapatric boundary estimation is based on males only.

**Figure:**
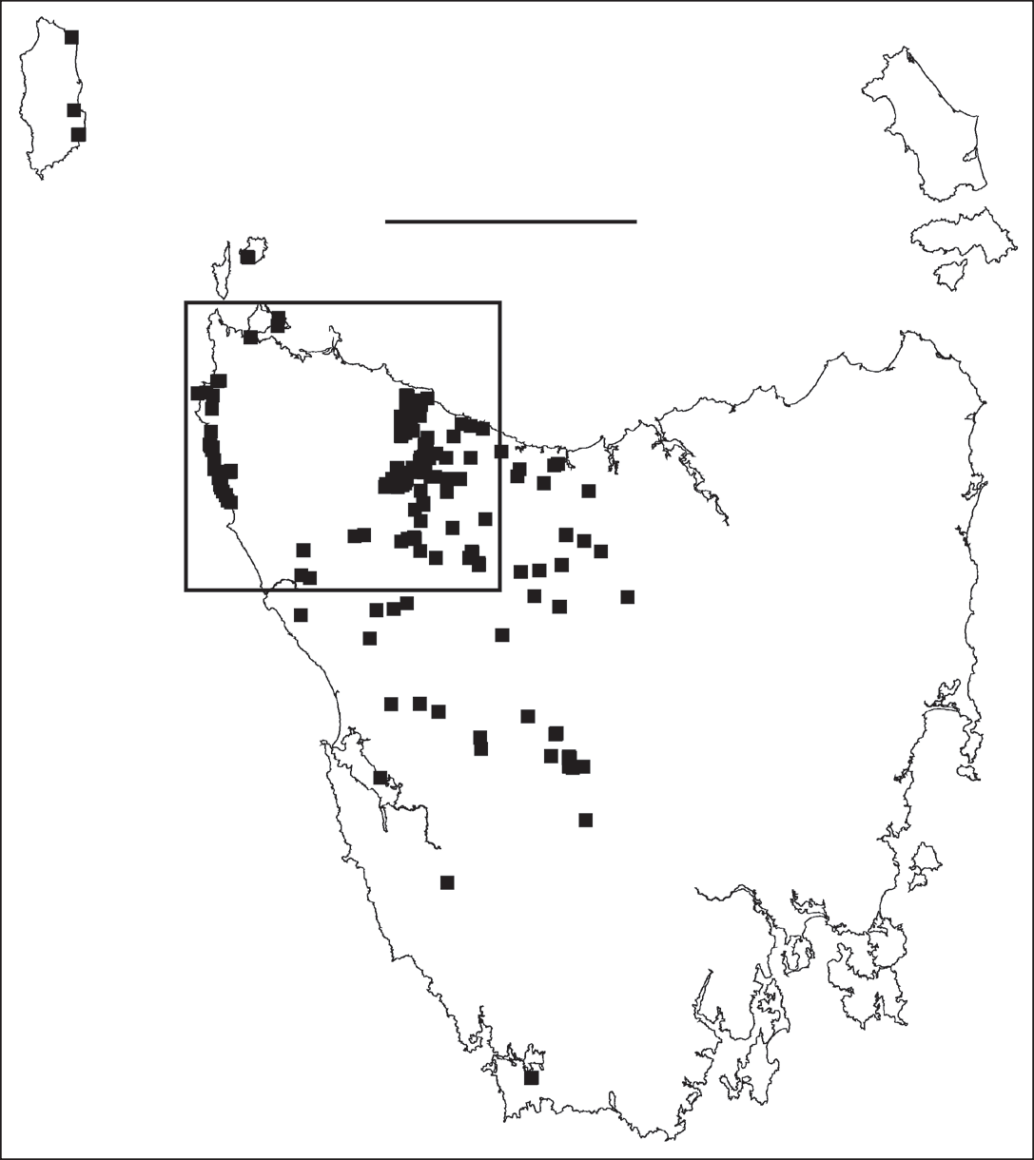
**Figure 1.** Localities for male *Tasmaniosoma hickmanorum*. Scale bar = 100 km. Rectangle indicates map extent in Figs 2–6.

Colour in the two species fades quickly in alcohol. Preserved females cannot be identified by colour after a few months, and long-preserved museum specimens can only be identified by examining the gonopods of mature males.

### Millipede sampling

For the present study I collected *Tasmaniosoma compitale* and *Tasmaniosoma hickmanorum* by hand during the daytime at 351 sites in northwest Tasmania between July 2009 and August 2011. Adults are night-wandering and can be found sheltering during the day under loose fragments of bark on dead tree stems and branches, under flat pieces of bark and woody litter on the ground, and within narrow, curled lengths of fallen bark from eucalypt crown branches. At most sites adults were collected within 30 minutes, but at sites where *Tasmaniosoma* spp. were uncommon I extended the search to ca 1 hour. Several sites were searched on more than one field day before *Tasmaniosoma* spp. were found, and a number of specimens were kindly provided by other collectors during the study period. All specimens were preserved in 80% ethanol and deposited as registered lots in the Queen Victoria Museum and Art Gallery, Launceston, Tasmania.

Sites were located in the field with a handheld GPS unit, or later the same day by reference to Google Earth. Because I collected specimens over an area up to ca 1 ha at each site, the uncertainty associated with each locality was recorded as ±25 m or ±50 m as appropriate.

**Note:** All known specimen records for *Tasmaniosoma compitale* and *Tasmaniosoma hickmanorum* are listed in the Appendix. The reader should refer to this Appendix for locations of placenames and other geographic features mentioned in the Results and Discussion sections. Site locations can be plotted from Appendix data either as UTM grid references or as latitude/longitude in GIS, or in Google Earth or other spatial data browsers using the included KML files.

### Boundary estimation

A parapatric boundary can be marked on a map by drawing a line equidistant from localities for each of the two species near the boundary. A ‘halfway between’ line of this kind is an appropriate estimator of the true location of the boundary if sampling localities are few and the map scale is coarse.

The present study generated hundreds of species localities and I was interested to trace the path of the main parapatric boundary (see below) at a scale appropriate to the spatial intensity of the sampling. I first generated a Delaunay network for male localities in the area of interest using the Delaunay triangulation tool in Quantum GIS version 1.4.0 (http://qgis.org/); see Fig. 4 and its caption for more details. I then deleted from the Delaunay polygon shapefile all triangles which had the same species at all three vertices. For clarity, I also deleted triangles running seawards from the two coastal ends of the boundary. The remaining set of 163 polygons (Fig. 5; see also its caption) consisted of triangles with one species at two of the vertices and the other species at the third. Finally, I generated centroids for the 163 triangles (Fig. 6) using the freeware Center of Mass extension (Jenness Enterprises, Flagstaff AZ, USA, http://www.jennessent.com/) for ArcView 3.2 GIS (ESRI, Redlands CA, USA).

Each of the 163 centroids is a point estimate for the location of the parapatric boundary. Where sampling localities are close together, the centroids approximate a line; see the eastern section of the boundary in Fig. 6. Where sampling localities are far apart, the centroids are diffusely distributed. The method used here is similar to triangulation wombling for scattered point data (Fortin and Drapeau 1995), but tentative ‘boundary elements’ have not been generated by joining centroids. The ‘cloud of centroids’ in Fig. 6 shows the relative uncertainty in estimating the location of the boundary from place to place, something which a hand-drawn or computed line cannot easily do.

### Spatial data sources

The GIS layers used in Fig. 7 for elevation contours, major streams, generalised geology and rainfall isohyets were sourced from the spatial data library of the Tasmanian state government (http://www.thelist.tas.gov.au) through the Queen Victoria Museum and Art Gallery.

## Results

### Millipede sampling overview

Adult *Tasmaniosoma* spp. were collected in every month of the year during the study period, but were much harder to find in the Tasmanian summer, December to March.

In northwest Tasmania, *Tasmaniosoma compitale* and *Tasmaniosoma hickmanorum* appear to be most abundant in natural forest and woodland of the regional *Eucalyptus* species, namely *Eucalyptus brookeriana*, *Eucalyptus delegatensis*, *Eucalyptus nitida*, *Eucalyptus obliqua*, *Eucalyptus ovata* and *Eucalyptus viminalis*. Both species can also be found in riparian or fire-sere stands of *Acacia dealbata* and *Acacia melanoxylon*. Both millipede species occur in eucalypt communities ranging from open forest with an iris ground layer (*Diplarrena* sp.), to regrowth forest with a dense understorey of small trees (*Pomaderris apetala* or *Leptospermum* spp.), to old-growth forest with an understorey of mature rainforest tree species (*Nothofagus cunninghamii*, *Phyllocladus aspleniifolius*, *Atherospermum moschatum*). Neither species is easy to find in old-growth *Nothofagus cunninghamii* rainforest or in heathland.

Populations of both *Tasmaniosoma compitale* and *Tasmaniosoma hickmanorum* appear to be tolerant of major habitat disturbance, including intense wildfire and clearfelling followed by burning and reafforestation. Both species are abundant in some plantations of *Eucalyptus globulus*, *Eucalyptus nitens* and *Pinus radiata*, especially first-rotation plantations established on former native forest sites and plantations closely bordering native forest, including narrow remnant strips close to watercourses. Both species are also occasionally found in small (<0.1 ha) vegetation remnants on cleared farmland, e.g. an isolated eucalypt copse or a riparian strip of *Acacia melanoxylon* over blackberry (*Rubus fruticosus*) and other weed species.

I was unable to find either species in certain apparently suitable habitat patches, despite repeated searches during the two-year study period. These distribution gaps were mainly clustered in the southeast of the study area near Waratah and Guildford, but were also close to former *Poa* grassland/woodland sites near Oonah. Populations of *Tasmaniosoma compitale* or *Tasmaniosoma hickmanorum* were found at sites within a few kilometres of the gaps.

### Main parapatric boundary

*Tasmaniosoma hickmanorum* is widespread in western Tasmania but there is a ca 4000 km^2^ gap in its distribution in the northwest (Figs 1, 2). The gap is filled by the distribution of *Tasmaniosoma compitale* (Fig. 3). The main parapatric boundary between the two species is ca 230 km long (Fig. 6) and has been closely mapped along Jefferson Road southeast of Preolenna and Rebecca Spur 3 southeast of Temma. At these two locations the boundary is ca 100 m wide (Fig. 8). The boundary appears to be just as narrow along Leonards Road south of Henrietta, Lyons Road south of Lapoinya, the Murchison Highway southeast of Waratah and at the north end of Hellyer Gorge, Oonah Road west of Oonah and Robbins Island Road north of West Montagu. At these locations I was unable to collect enough males during the study period for reliable fine-scale mapping.

**Figure:**
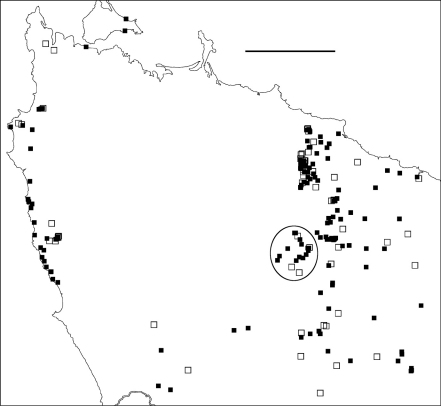
**Figure 2.** Localities for *Tasmaniosoma hickmanorum* males (filled squares) and females identified by colour (open squares). Ellipse encloses Arthur-Hellyer ‘island’ (see text for explanation). Scale bar = 25 km.

**Figure:**
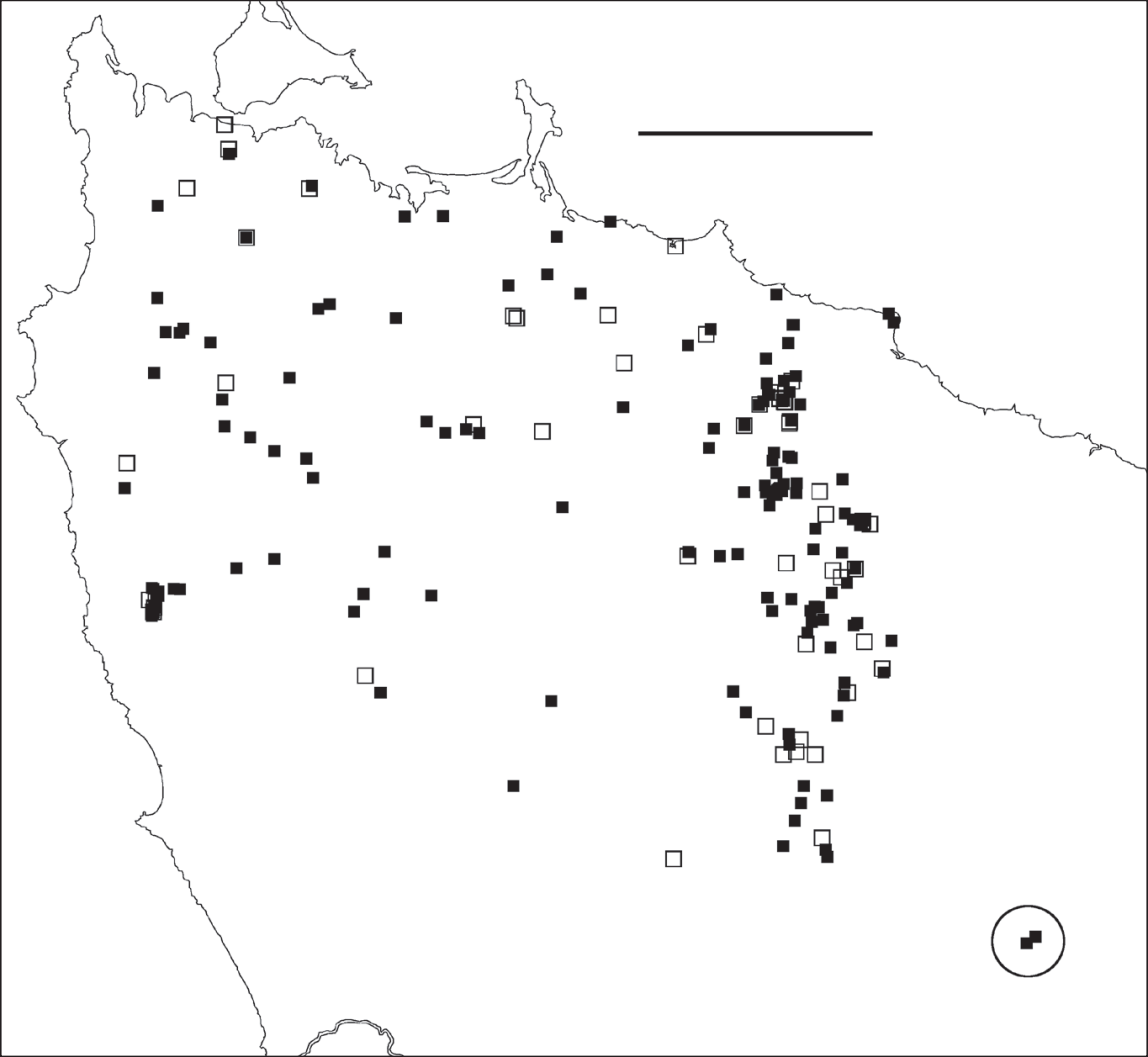
**Figure 3.** Localities for *Tasmaniosoma compitale* males (filled squares) and females identified by colour (open squares). Outliers (circled) are at the Vale of Belvoir. Scale bar = 25 km.

Boundary mapping is no longer possible near Wynyard, Redpa and other farming localities in northwest Tasmania. *Tasmaniosoma compitale* and *Tasmaniosoma hickmanorum* populations persist in native vegetation remnants in these areas, but are separated by intensively managed pasture and cropland. Mapping is also difficult along the southern and southwestern sections of the main parapatric boundary, which traverses unroaded, largely wild country.

### Arthur-Hellyer ‘island’

A ca 40 km^2^ ‘island’ of *Tasmaniosoma hickmanorum* localities occurs in the upper reaches of the Arthur and Hellyer Rivers near Hellyer Gorge (Fig. 2), apparently enclosed by *Tasmaniosoma compitale* localities. The parapatric boundary of this ‘island’ has not yet been closely mapped on its eastern and northern sides, and the western and southern sections of the boundary (not yet located) are in unroaded, steeply dissected and thickly vegetated terrain.

### Possible translocations

Males of *Tasmaniosoma compitale* were found ca 20 km within the *Tasmaniosoma hickmanorum* range at the Vale of Belvoir (Fig. 3). It is not yet known whether this is a naturally isolated occurrence or the result of translocation. The ca 500 ha of open grassland at the Vale has been grazed by cattle during the summer months for more than 100 years, and *Tasmaniosoma compitale* may have been unintentionally introduced into the area by cattle-carrying trucks.

Translocations of *Tasmaniosoma compitale* over shorter distances may also have occurred in intensively managed forest close to the main parapatric boundary. For example, I collected females of this species at three neighbouring sites along Ten Foot Track off Preolenna Road. The three sites are surrounded by *Tasmaniosoma hickmanorum* occurrences and are close to a logging road used by log-carting trucks in recent years.

### Co-occurrence

At two sites along the main parapatric boundary (Figs 4-6) I found males of *Tasmaniosoma compitale* and *Tasmaniosoma hickmanorum* within a few metres of each other, on Leonards Road south of Henrietta and on Talunah Road west of Hampshire. At another 11 sites close to the main parapatric boundary I found possible co-occurrences, but at least one species was represented only by females identified by colour. At two of the latter sites, both species were found sheltering under loose bark on the same fallen tree.

**Figure:**
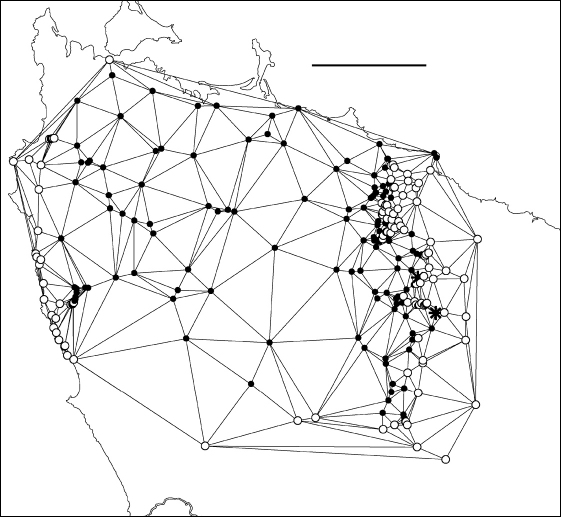
**Figure 4.** Delaunay triangulation of localities for male *Tasmaniosoma compitale* (filled circles), *Tasmaniosoma hickmanorum* (open circles) and co-occurrences (stars). The set of localities used has been trimmed to the vicinity of the parapatric boundary, and *Tasmaniosoma hickmanorum* localities in the Arthur-Hellyer ‘island’ and *Tasmaniosoma compitale* localities at the Vale of Belvoir have been excluded. Scale bar = 25 km.

**Figure:**
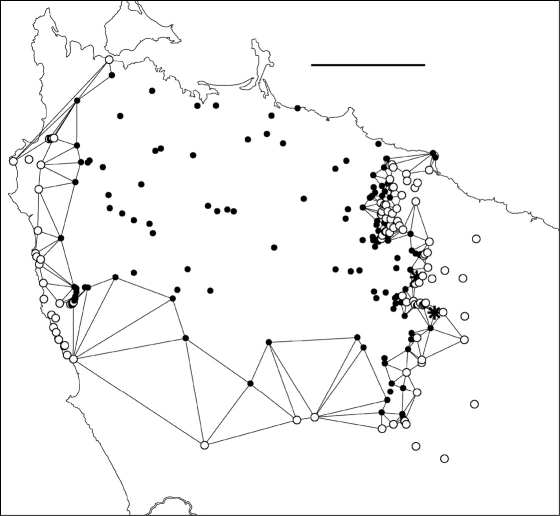
**Figure 5.** Edited Delaunay triangulation of localities for male *Tasmaniosoma compitale* (filled circles), *Tasmaniosoma hickmanorum* (open circles) and co-occurrences (stars); see text for explanation. Scale bar = 25 km.

**Figure:**
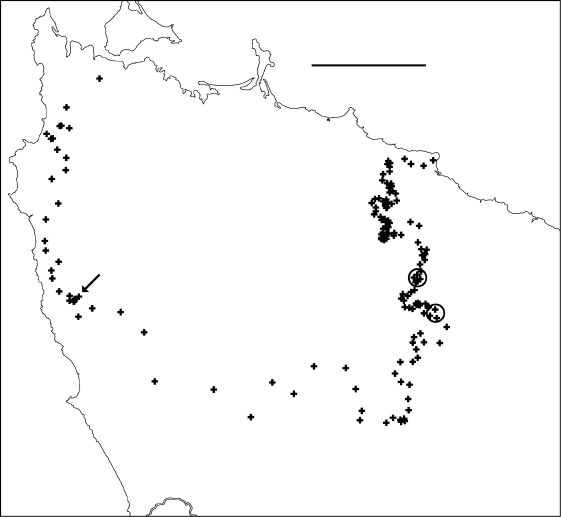
**Figure 6.** Centroids of triangles in Figure 5. Co-occurrences of *Tasmaniosoma compitale* and *Tasmaniosoma hickmanorum* males are circled. Arrow points to area shown in Figure 8. Scale bar = 25 km.

### No evidence of hybrids

None of the males collected near the main parapatric boundary or the boundary of the Arthur-Hellyer ‘island’ had gonopods intermediate between *Tasmaniosoma compitale* and *Tasmaniosoma hickmanorum*, or were intermediate in live colouring.

### Environmental incongruence

As shown in Fig. 7A, the main parapatric boundary rises from the north coast near Robbins Island (western section of the boundary) to 600-700 m at its southeast corner, then returns to the coast near Table Cape, ca 75 km from its starting point. It crosses most of the west coastal rivers north of Sandy Cape and the headstreams of both the major inland river systems in the region (Arthur and Pieman) before descending to the north coast; the northeast section of the boundary more or less follows the Flowerdale River (Fig. 7B). The parapatric boundary crosses numerous geological boundaries (Fig. 7C) and its eastern section runs along a fairly steep rainfall gradient (Fig. 7D), i.e. at right angles to isohyets.

Both *Tasmaniosoma compitale* and *Tasmaniosoma hickmanorum* occur in a wide range of vegetation types close to the parapatric boundary. If there are major environmental boundaries which are spatially congruent with the biogeographical one separating the two species, they are not obviously related to topography, geology, climate or vegetation.

**Figure:**
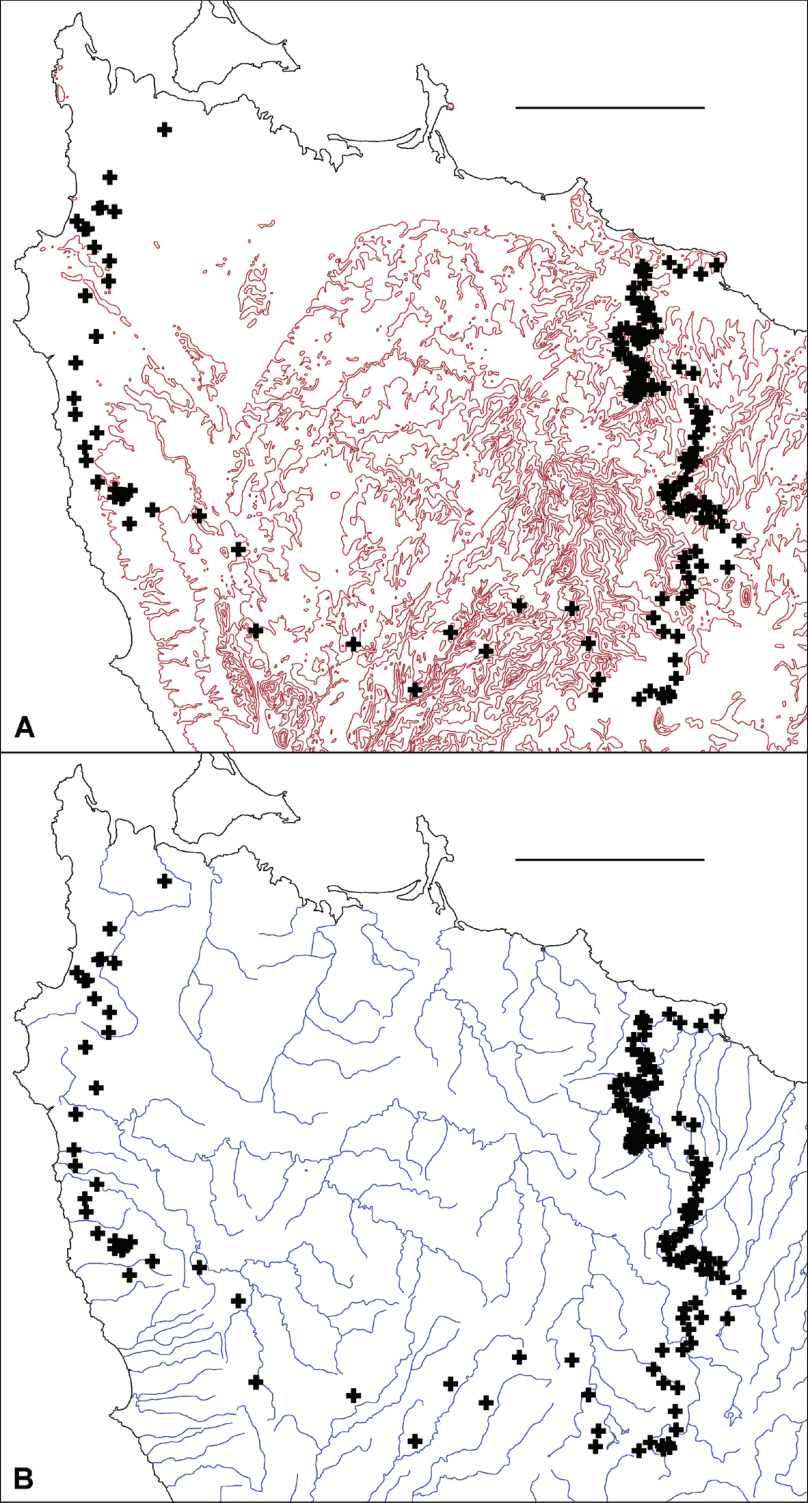
**Figure 7A, B.** Centroids from Fig. 6 superimposed on 100 m elevation contours **A** and principal rivers **B**. Scale bars = 25 km.

**Figure:**
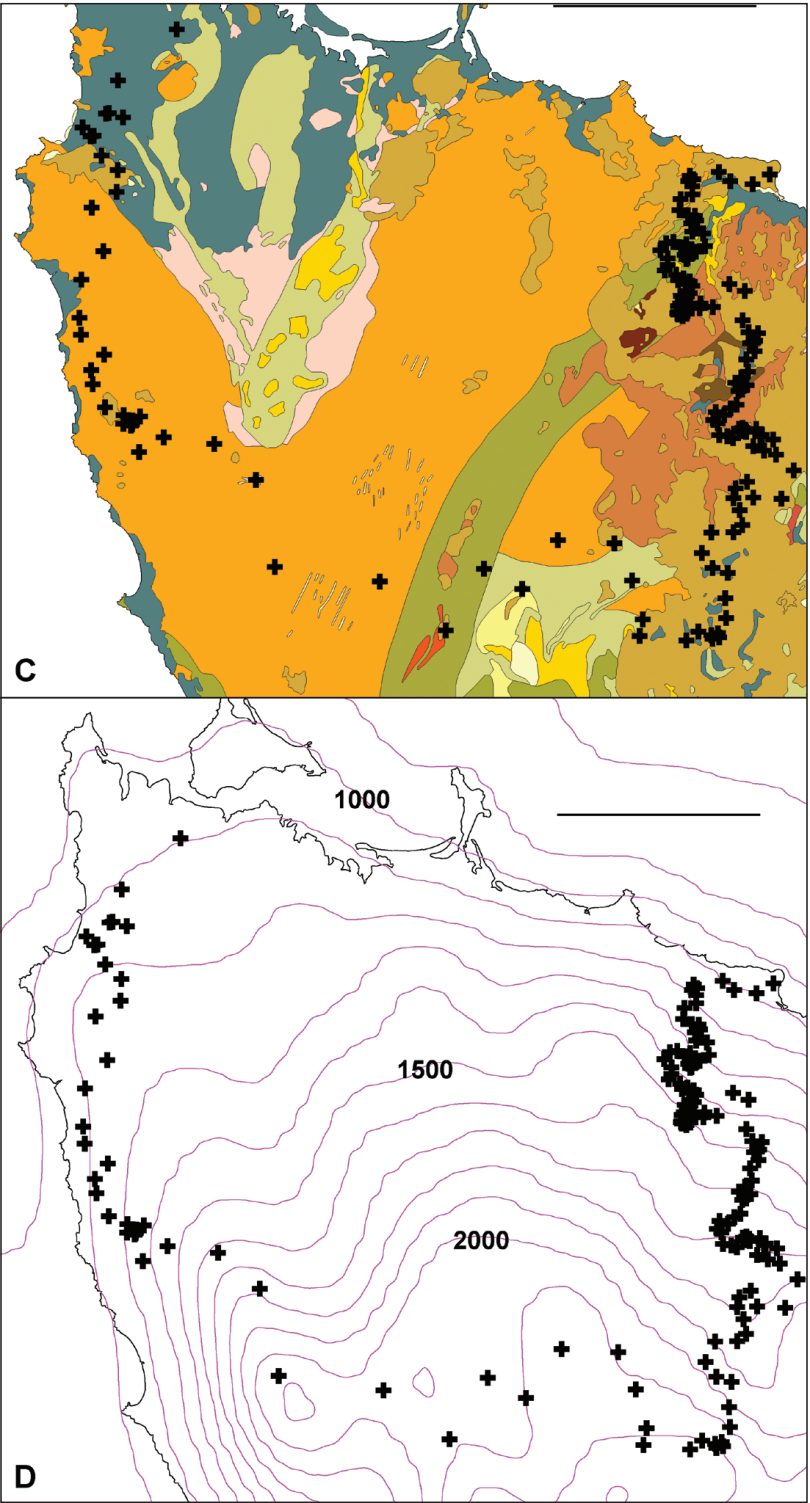
**Figure 7C, D.** Centroids from Fig. 6 superimposed on simplified bedrock geology **C** and mean annual rainfall isohyets, in mm **D**. In **C**, colours crossed by main parapatric boundary represent (anticlockwise from top left) Quaternary coastal sand and gravel (gray-blue), Precambrian siltstone and mudstone (orange), Precambrian metamorphics (green), Cambrian conglomerate and siltstone (gray-green), Tertiary basalt (light brown) and Permian glaciomarine sedimentary rocks (red-brown). Scale bars = 25 km.

## Discussion

The aim of this study was to map the boundary between the *Tasmaniosoma compitale* and *Tasmaniosoma hickmanorum* distributions as a knowledge base for future studies of parapatry and speciation. The parapatric boundary between these two species is the longest and narrowest I am aware of in the Australian millipede fauna. It is particularly well-suited to field study because much of it is easily accessed by all-weather roads, and because sections of the boundary run through little-disturbed tracts of native vegetation, including primary forest. The two millipede species are ecologically resilient and can be very abundant in eucalypt forest at lower elevations.

Several of the the largest and least disturbed patches of forest along the boundary are on public land managed by Forestry Tasmania, a government-owned forestry business. One such patch covers ca 12 km^2^ on the upper Flowerdale River between Lapoinya and Preolenna Roads, on the eastern arm of the boundary. The forest is a mosaic of formal and informal reserves, production forest and plantation, with privately owned forest on the periphery. A similar but smaller mosaic is found along Rebecca Spur 3 and south of the Rebecca Road – Rebecca Spur 3 junction (Fig. 8) on the western arm of the boundary.

**Figure:**
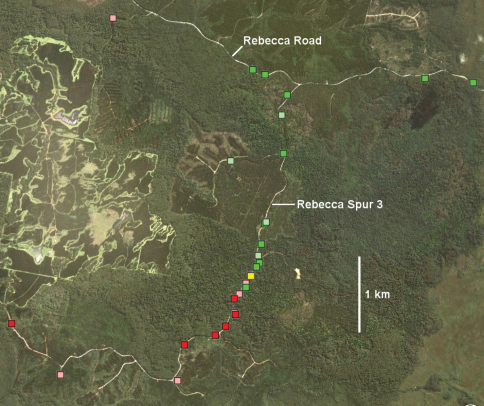
**Figure 8.** Rebecca Spur 3 area (marked with arrow in Fig. 6) in Google Earth image dated 11 October 2010. Markers: *Tasmaniosoma compitale* males (darker green) and females identified by colour (lighter green), *Tasmaniosoma hickmanorum* males (darker red) and females identified by colour (lighter red), and possible co-occurrence of females identified by colour (yellow).

The parapatric boundary also crosses private land. Because the future of privately owned forest is less certain in Tasmania than that of formally reserved forest on public land, future field studies on suitable private blocks might be given a high priority. I was allowed access during the study period to a large private block near Henrietta, just north of the Takone Road. The Henrietta block carries even-aged eucalypt regrowth and populations of both *Tasmaniosoma compitale* and *Tasmaniosoma hickmanorum*.

An unanswered question is whether the two millipede species are well-distributed across heathland near the west coast. *Tasmaniosoma hickmanorum* is abundant in shrubby coastal vegetation, and to the east *Tasmaniosoma compitale* has been found close to scattered, small eucalypts in heath. As currently estimated (Fig. 6), the main parapatric boundary crosses ca 50 km of heathland in the formally reserved Arthur-Pieman Conservation Area.

## Appendix

Specimen records as of 8 August 2011 for *Tasmaniosoma compitale*, *Tasmaniosoma hickmanorum* and unassigned females of these species. doi: 10.3897/zookeys.156.1893.app

**Description.** Complete records are given in the annotated CSV file ‘Parapatry_records.csv’. The compressed file ‘Parapatry_KML.zip’ contains separate KML files for males of the two species, females and juveniles of the two species (identified by colour), co-occurrences, and females not assignable to either species. Each KML record has an associated museum registration number and users should refer to ‘Parapatry_records.csv’ for more information on that record.

## References

[B1] Fortin M-J Drapeau P (1995) Delineation of ecological boundaries: comparison of approachesand significance tests. Oikos 72: 323-332 doi: 10.2307/3546117

[B2] Mesibov R (2003a) A new genus of Tasmanian millipedes (Diplopoda, Polydesmida, Dalodesmidae) with unusual spiracles and a mosaic distribution. Memoirs of Museum Victoria 60 (2): 197-206

[B3] Mesibov R (2003b) The millipede genus *Gasterogramma* (Diplopoda: Polydesmida: Dalodesmidae) in Tasmania, Australia, with descriptions of seven new species. Memoirs of Museum Victoria 60 (2): 207-219

[B4] Mesibov R (2003c) Lineage mosaics in millipedes. African Invertebrates 44 (1): 203-212

[B5] Mesibov R (2004) A new genus of millipedes (Diplopoda: Polydesmida: Dalodesmidae) from Tasmania, Australia with a mosaic distribution. Zootaxa 480: 1-23

[B6] Mesibov R (2006) The millipede genus *Lissodesmus* Chamberlin, 1920 (Diplopoda: Polydesmida: Dalodesmidae) from Tasmania and Victoria, with descriptions of a new genus and 24 new species. Memoirs of Museum Victoria 62 (2): 103-146

[B7] Mesibov R (2010) The millipede genus *Tasmaniosoma* Verhoeff, 1936 (Diplopoda, Polydesmida, Dalodesmidae) from Tasmania, Australia, with descriptions of 18 new species. ZooKeys 41: 31-80 doi: 10.3897/zookeys.41.420 10.3897/zookeys.488.9460 PMC4389123 25878522

[B8] Shelley RM (1990a) Revision of the milliped family Eurymerodesmidae (Polydesmida: Chelodesmidea). Memoirs of the American Entomological Society 37: 1-112

[B9] Shelley RM (1990b) Are allopatric/parapatric mosaic complexes widespread in the Diplopoda? In: Minelli A (Ed) Proceedings of the 7th International Congress of Myriapodology. EJ Brill, Leiden, 2 3.

